# Proteomic analysis of *Fusarium oxysporum* f. sp. *cubense* tropical race 4-inoculated response to Fusarium wilts in the banana root cells

**DOI:** 10.1186/1477-5956-11-41

**Published:** 2013-09-26

**Authors:** Xingshen Li, Tingting Bai, Yunfeng Li, Xiaolei Ruan, Huaping Li

**Affiliations:** 1 State Key Laboratory of Conservation and Utilization of Subtropical Agro-bioresources, Guangzhou, Guangdong 510642, China; 2 College of Natural Resources and Environment, South China Agricultural University, Guangzhou, 510642, China

**Keywords:** Banana, *Fusarium oxysporum* f. sp. *cubense* tropical race 4, Root proteome, Induced resistance, Two-dimensional electrophoresis

## Abstract

**Background:**

Fusarium wilt of banana is one of the most destructive diseases in the world. This disease has caused heavy losses in major banana production areas. Except for molecular breeding methods based on plant defense mechanisms, effective methods to control the disease are still lacking. Dynamic changes in defense mechanisms between susceptible, moderately resistant, and highly resistant banana and *Fusarium oxysporum* f. sp. *cubense* tropical race 4 (Foc4) at the protein level remain unknown. This research reports the proteomic profile of three banana cultivars in response to Foc4 and transcriptional levels correlated with their sequences for the design of disease control strategies by molecular breeding.

**Results:**

Thirty-eight differentially expressed proteins were identified to function in cell metabolism. Most of these proteins were positively regulated after Foc4 inoculation. These differentially regulated proteins were found to have important functions in banana defense response. Functional categories implicated that these proteins were associated with pathogenesis-related (PR) response; isoflavonoid, flavonoid, and anthocyanin syntheses; cell wall strengthening; cell polarization; reactive oxygen species production and scavenging; jasmonic acid-, abscisic acid-, and auxin-mediated signaling conduction; molecular chaperones; energy; and primary metabolism. By comparing the protein profiles of resistant and susceptible banana cultivars, many proteins showed obvious distinction in their defense mechanism functions. PR proteins in susceptible ‘Brazil’ were mainly involved in defense. The proteins related to PR response, cell wall strengthening and antifungal compound synthesis in moderately resistant ‘Nongke No.1’ were mainly involved in defense. The proteins related to PR response, cell wall strengthening, and antifungal compound synthesis in highly resistant ‘Yueyoukang I’ were mainly involved in defense. 12 differentially regulated genes were selected to validate through quantitative real time PCR method. Quantitative RT-PCR analyses of these selected genes corroborate with their respective protein abundance after pathogen infection.

**Conclusions:**

This report is the first to use proteomic profiling to study the molecular mechanism of banana roots infected with Foc4. The differentially regulated proteins involved in different defense pathways are likely associated with different resistant levels of the three banana cultivars.

## Background

*Musa spp.* is one of the most important economic and agricultural crops in the world. However, the annual production and attribute of banana are greatly reduced by various infectious diseases caused by fungi, bacteria, and viruses. Among these diseases, the Fusarium wilt of banana (a.k.a. panama disease) is the most important lethal disease; this disease is caused by the soil-borne fungus *Fusarium oxysporum* f. sp*. cubense* (E.F. Smith) Snyder and Hansen (*F. oxysporum*) [1,2]. The disease has caused severe yield losses in all banana-producing areas in Asia, Africa, Australia, and the tropical Americas [3]. Furthermore, many planting areas are sharply declining because of *F. oxysporum* chlamydospores, which enable the fungi to persist in soil even under bad conditions and even in the absence of the host. Once soil is infected with *F. oxysporum*, susceptible cultivars can hardly be successfully replanted in the same land for the span of 30 years [4]. Currently, *F. oxysporum* is classified into four races based on pathogenic characterization on different banana cultivars. Among the four races of *F. oxysporum*, race 4 has been thought to be a major threat to the production of banana because it can affect almost all cultivars, except those infected with tropical race 1 and tropical race 2 [5].

*F. oxysporum* infection is divided into several steps: recognition of roots, attachment and colonization of root surface, penetration and colonization of root cortex, and hyphal proliferation of xylem vessels [6]. Particularly, the germination of fungal spores in soil is important in the entire process, which relies on the exudates of banana roots [7]. Therefore, roots are important for infection completion and plant growth because they supply nutrients for fungal proliferation and assimilate water and nutrients, respectively. In the present study, roots were the main organ used for the investigations. To date, methods for controlling the disease include only physical and chemical measures, all of which are ineffective because the spread of the disease in the world has not been suppressed. Therefore, the development of resistant cultivars through molecular breeding based on plant defense mechanisms is urgently needed, besides biological control of Fusarium wilt disease as one of implemented disease management was considered [8].

During the course of evolution, plants have developed an innate immune defense system against various pathogens [9]. The initiation of recognition of pathogen-associated molecular pattern (PAMP)-triggered immunity (PTI) is the first branch of plant immunity, which relies on PAMP patterns by pattern-recognition receptors (PRRs) at the cell surface [10]. During the course of PTI, several intracellular responses are associated with plant defense, including changes in Ca^2+^ flux, reactive oxygen species (ROS) and phytoalexin production, mitogen-activated protein kinase cascades, plant cell wall reinforcement at infection sites, and stomatal closure [11,12]. Pathogens have successfully evolved strategies to suppress PTI by secreting effector proteins directly into the plant cell to enhance virulence [13]. However, some of these effectors are recognized by plant resistance (R) proteins in the second branch of plant immune system to activate effector-triggered immunity (ETI) [14]. Generally, ETI is similar to PTI, although the former is believed to be stronger and faster than the latter, which is often accompanied by hypersensitive response/localized cell death [15]. In addition, the plant immune system is regulated by hormone signaling molecules, such as salicylic acid (SA), jasmonic acid (JA), ethylene (ET), auxins, gibberillins, abscisic acid (ABA), cytokinins, brassinosteroids, and peptide hormones [14,16]. Among these molecules, SA, JA, and ET are considered important in plant responses to abiotic and biotic stresses [11]. For example, the JA/ET pathway is involved in defense response to necrotrophic pathogens, herbivorous and wounding in *Arabidopsis thaliana*[17]. The ABA pathway has a crucial function in defense responses to drought, low temperature, salinity, and pathogens [18]. The auxin pathway has a significant function in defense responses to bacteria, necrotrophic fungi, and oomycete pathogens in *A. thaliana*; in addition, the interplay of auxin and antimicrobial secondary metabolites are involved in the same tryptophan pathway [19]. Antimicrobial secondary metabolites are mainly composed of phenolic compounds that are produced in the phenylpropanoid pathway to form lignins, lignans, neolignans, flavonoids, and anthocyanins [20].

Banana is an important monocot crop species, and the discovery of its defense mechanism is a prerequisite for disease control through molecular breeding. To date, research on the defense mechanism of banana against *F. oxysporum* is insufficient. Several defense-associated genes were identified in resistant banana roots by suppression subtractive hybridization [21]. Through transcriptomic profile analysis, Li et al. [22] found that the recognition of PAMPs and defense-related genes may contribute to Foc4 resistance in banana. With the completion of banana genome sequencing, proteomic profile analysis can provide a powerful tool to investigate the complex defense mechanism in banana [23]. In recent years, several proteomic studies have been successfully applied to investigate the effect of cold tolerance and osmotic stresses on banana growth and development [24,25]. However, proteomic profiling of banana roots in response to *F. oxysporum* has yet to be performed.

The present study investigated the resistance mechanism of banana roots, revealing the dynamics of defense against *F. oxysporum* infection. The defense-related genes involved in susceptible and resistant cultivars were identified using two-dimensional electrophoresis (2-DE). A total of 43 differentially expressed proteins in pathogen-challenged banana roots were identified. These dynamic proteins were found associated with defense. They were involved in the recognition of pathogen signal transduction, the production of ROS and antifungal compounds, the expression of PR genes, and the reinforcement of cell walls. This report is the first to use proteomic profiling to study the molecular mechanism of banana roots infected with Foc4. This study revealed the dynamic changes in banana roots infected with Foc4. Our results provided insights into the defense mechanisms of banana and may serve as a basis for the control of *Fusarium* wilt.

## Results

### Effect of Foc4 treatment on banana roots

**Figure 1:**
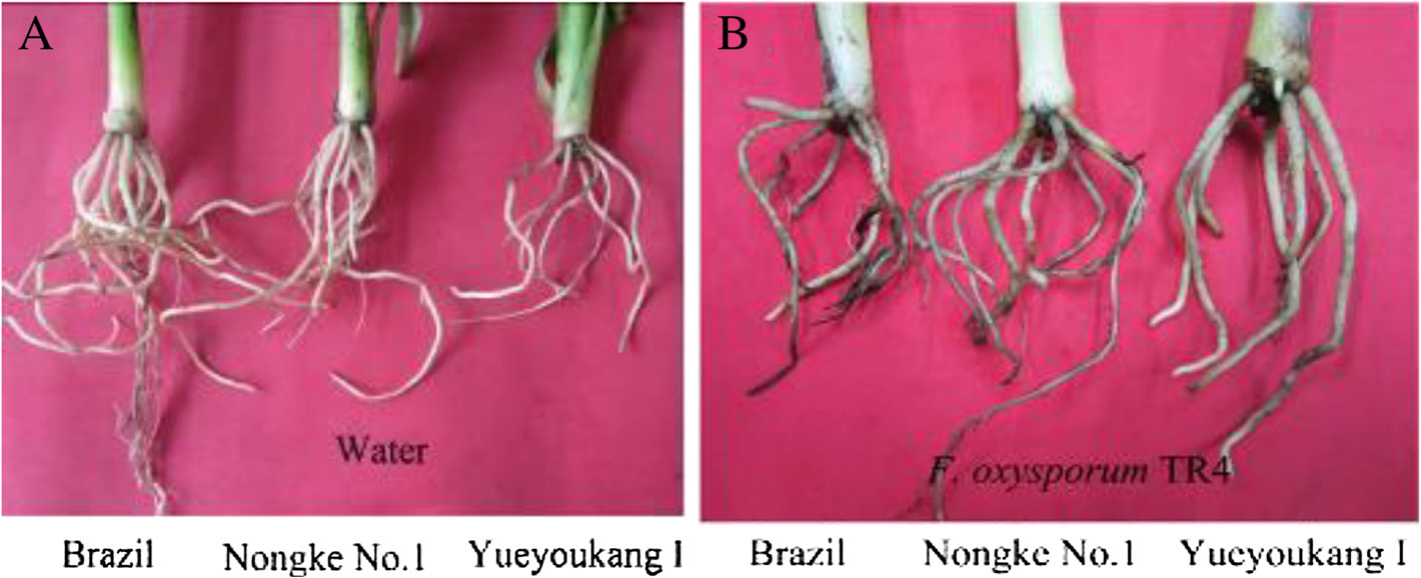
**Disease symptoms from *Musa *roots with treatment of *F. oxysporum*. A**: Banana roots/H_2_O control, and **B**: Banana roots/infection. Susceptible ‘Brazil’, moderately resisitant ‘Nongke No.1’ and highly resistant ‘Yueyoukang I’ were treated at 3D after *F. oxysporum* inoculation, compared with water-treated control.

In this study, we compared injury symptoms of roots in response to Foc4 infection in three banana cultivars (Figure 1). At 3 d after inoculation, no significant injury in the roots of highly resistant ‘Yueyoukang I’ was observed, and many progressively smaller lateral roots grew from the main roots. Less necrotic symptoms occurred in the roots of moderately resistant ‘Nongke No.1’ , and only a few lateral roots grew from the main roots. However, Foc4-susceptible ‘Brazil’ displayed severe necrotic symptoms in the roots.

### 2-DE analysis of pathogen-induced proteins in banana roots

**Figure 2:**
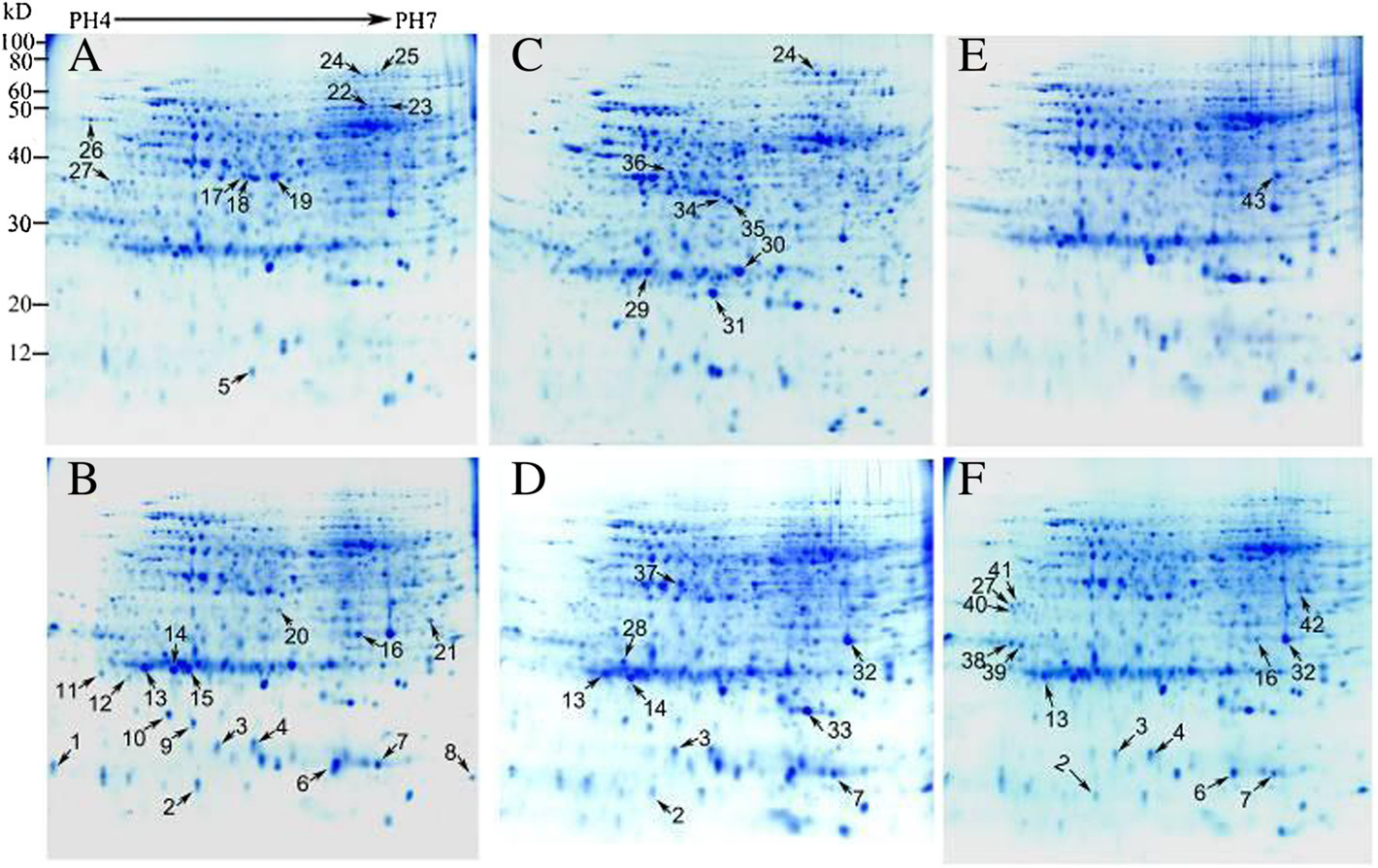
**Representative 2-DE of proteins from Musa roots with treatment of *F. oxysporum*. A**, Brazil/H_2_O control; **B**, Brazil/infection; **C**, Nongke No.1/ H_2_O control; **D**, Nongke No.1/infection; **E**, Yueyoukang I/ H_2_O control; **F**, Yueyoukang I/infection. Proteins (550 μg) were loaded on an 18 cm IPG strip (pH 4–7) for IEF, following electrophoresis of 12% SDS-PAGE and CBB G-250 staining. The different expression spots present at the position of arrows are compared with their respective controls.

**Figure 3:**
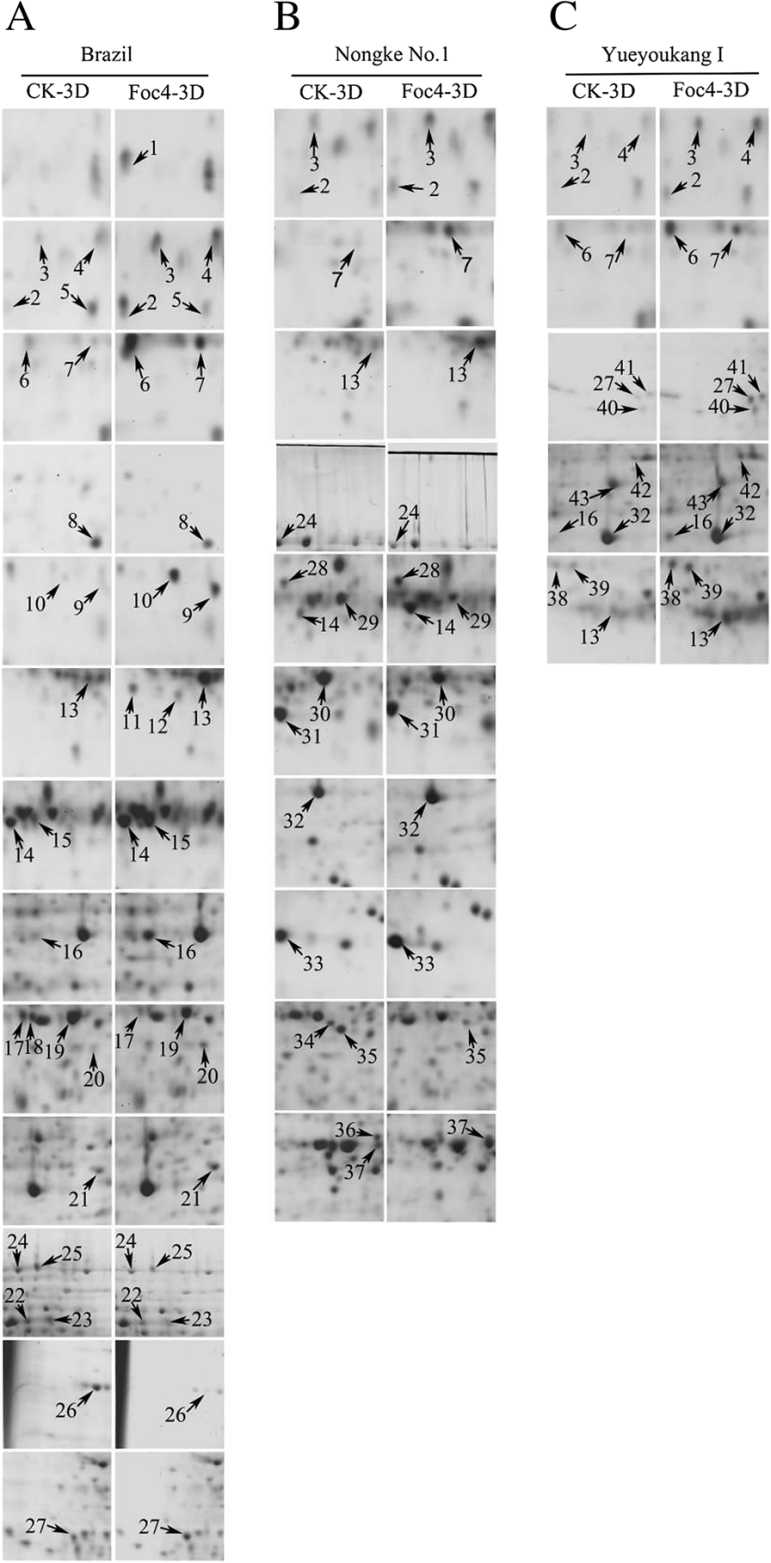
**2-DE analysis of differentially expressed protein spots in banana roots with *F. oxysporum *treatment. A**: Brazil, **B**: Nongke No.1, and **C**: Yueyoukang I. Each sample had three biological replicates. Arrows indicate the abundance change of the identified proteins.

To investigate the proteomic changes in banana roots in response to Foc4, we performed protein profile analysis on three banana cultivars, namely, susceptible ‘Brazil’ , moderately resistant ‘Nongke No.1’ , and highly resistant ‘Yueyoukang I’. Banana roots at the fourth leaf stage were sampled at the time points of 3D after Foc4 or H_2_O treatment. Subsequently, the total proteins of treated samples were extracted for 2-DE analysis using 18 cm gel strips. For each biological sample, proteomic profile analysis was conducted at least three times to obtain high reproducibility. All protein spots were distributed in molecular mass values ranging from 10 kDa to 100 kDa, with isoelectric point (PI) ranging from 4 to 7. Approximately 600 protein spots were reproducibly matched in Coomassie Brilliant Blue (CBB)-stained gels. A total of 58 protein spots exhibited significant differences (at least twofold changes) in abundance (Figure 2). Of these protein spots, 27 were detected in susceptible ‘Brazil’ , 16 were detected in moderately resistant ‘Nongke No.1’ , and 15 were detected in highly resistant ‘Yueyoukang I’ (Figure 3). Particularly, 7 separate protein spots were shared for any two cultivars, and 4 protein spots were shared for three cultivars (spot Nos. 2, 3, 7 and 13). Although most spots showed quantitative changes, 5 showed qualitative changes, of which 3 were only detected in infected susceptible ‘Brazil’ (spot Nos. 1, 11, and 12) and 2 were detected in control moderately resistant ‘Nongke No.1’ (spot Nos. 34 and 36). A total 53 spots (91%) showed similar variation patterns in three banana cultivars, of which 37 were up-regulated and 16 were down-regulated after the infection. Clearly, 27 spots showed up- or down-regulation in only one of the three banana cultivars, of which 14 (54%), 7 (58%), and 6 (40%) showed line-specific volume changes in susceptible ‘Brazil’ , moderately resistant ‘Nongke No.1’ , and highly resistant ‘Yueyoukang I’ , respectively. These results implied that the proteins might be independently or cooperatively involved in different cellular pathways in response to *F. oxysporum* infection in banana.

### Identification of differentially expressed proteins

To select defense proteins in banana roots that respond to Foc4, significant differential spots in CBB-stained gels were excised manually and subsequently analyzed by matrix-assisted laser desorption/ionization time-of-flight/time-of-flight (MALDI-TOF/TOF) mass spectrometer. A total of 43 spots were successfully identified by querying the *Musa acuminate* protein database (http://banana-genome.cirad.fr/content/download-dh-pahang), MASCOT NCBInr protein database and Plant_EST database (http://www.matrixscience.com/cgi/search_form.pl?FORMVER=2&SEARCH=SQ) using PI and molecular weight values (Additional file 1: Table S1). Among these protein spots, 37 were matched to the *M. acuminate* proteins, only 1 was matched to the Job’s Tears (*Coix lachryma-jobi* L.) protein, and 5 (spot Nos. 3, 4, 5, 6, and 7) were matched to *M. acuminate* expressed sequence tags (ESTs) without any functional information. Protein spot No. 35 is shown in Supporting Information Figure 4 as an example. Most protein spots are unique. However, more than one spot with only small shifts in molecular weight and/or pI were occasionally identified as the same sequence in the gel, which is considered as a common feature of 2-DE [26]. The protein spots were identified as glutamine synthetase nodule isozyme (spot Nos. 17 and 18), putative aconitate hydratase (spot Nos. 24 and 25), heat shock 22 kDa protein (spot Nos. 9 and 10), and Germin-like protein 12–1 (spot Nos. 11, 12, 13, and 15). The occurrence of protein shifts is explained as posttranslational modification or degradation.

**Figure 4:**
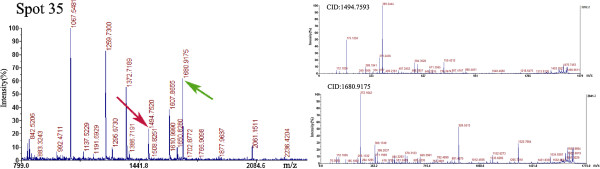
Identification of No. 35 protein spot from banana roots inoculated with Foc4 by MALDI-TOF/TOF MS.

**Figure 5:**
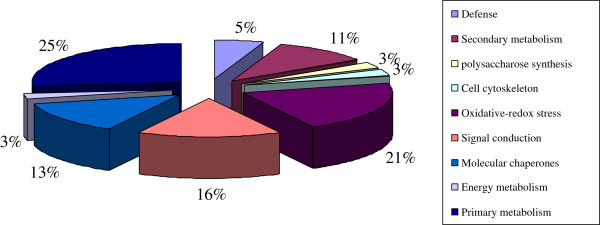
Functional category distribution of 38 identified proteins with known functions under the treatment with Foc4.

The predicted functions of the identified proteins were classified into nine categories based on the annotations from the *Musa acuminate* database, NCBInr database and the Gene Ontology database: defense (5%), secondary metabolism (11%), polysaccharose synthesis (3%), cell cytoskeleton (3%), oxidative-redox stress (21%), signal conduction (16%), molecular chaperones (13%), energy metabolism (3%), and primary metabolism (25%) (Figure 5, Additional file 1: Table S1). The protein identity of 14 up-regulated spots in susceptible ‘Brazil’ was determined: 2 are related to disease defense reactions, 3 are related to oxidative-redox stress, 2 are molecular chaperone proteins, 1 is related to energy, 2 are involved in primary metabolism, and 4 are ESTs. The protein identity of 9 up-regulated spots in moderately resistant ‘Nongke No.1’ was determined: 2 is an EST, 1 is a PR protein, 2 are related to oxidative-redox stress, 2 are involved in secondary metabolism, 1 is related to signal conduction, and 1 is related to cell polarization that is beneficial for defense. The protein identity of 14 up-regulated spots in highly resistant ‘Yueyoukang I’ was determined: 4 are ESTs, 2 are related to defense reactions, 1 is related to cell wall strengthening, 1 is a molecular chaperone, 1 is related to oxidative-redox stress, 2 are related to signal conduction, 2 are beneficial for primary metabolism, and 1 is involved in secondary metabolism. Overall, 23 up-regulated protein spots are associated with resistance or defense in the three banana cultivars, except for 4 ESTs. Among them, protein spot Nos. 2 and 16 are for basal resistance, spot Nos. 28 and 32 are for the production of lignin and antifungal compounds in secondary metabolism, spot No. 42 is for cell wall strengthening, spot No. 37 is for defense with cell polarization, spot Nos. 13, 14, and 15 are for the ROS production and scavenging system, spot Nos. 33, 38, and 39 are related to defense signal conduction, and spot Nos. 9, 10, and 40 are molecular chaperones in various stresses. These results implicated that antifungal secondary metabolism, lignin production, cell polarization, polysaccharose synthesis, and ABA defense signaling play key roles in the disease resistance mechanism of banana.

The protein identity of 10 down-regulated spots in susceptible ‘Brazil’ was determined: 6 are involved in primary metabolism, 2 are molecular chaperones, and 1 is a signal protein, except for 1 EST. The protein identity of 5 down-regulated spots in moderately resistant ‘Nongke No.1’ was determined: 3 are related to oxidative-redox stress, 1 is a signal protein, and 1 is related to the production of primary metabolites. Spot 43 is the only down-regulated spot in highly resistant ‘Yueyoukang I’ , and it is involved in primary metabolism. The protein identity of 15 down-regulated spots in all banana cultivars was determined: 7 is involved in primary metabolism, 2 are related to molecular chaperones, 2 are for signal conduction, and 3 are for antioxidant defense, except for 1 EST.

### Transcript analysis of differential proteins

**Figure 6:**
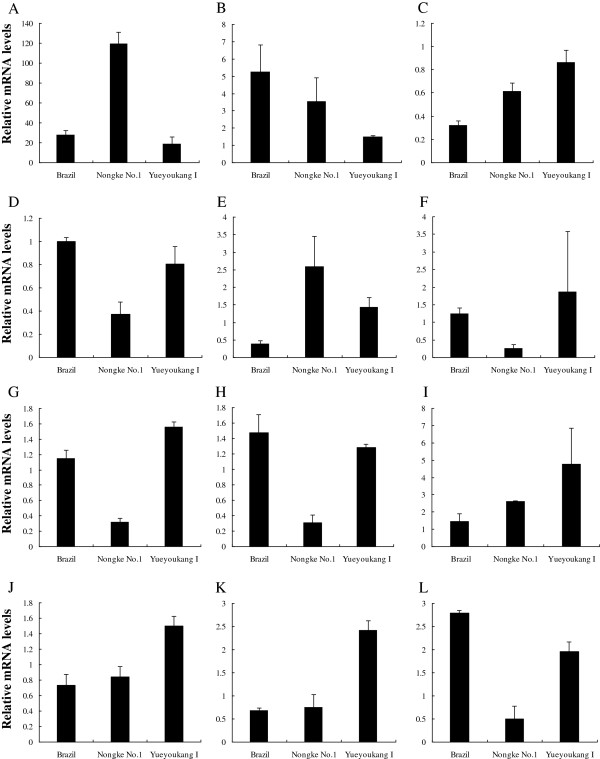
**Transcript analysis of 12 differentially expressed genes in response to Foc4 infection by qRT-PCR. A**: Pathogenesis-related protein 1, **B**: Protein IN2-1 homolog B, **C**: NADP-dependent malic enzyme, **D**: Protein disulfide-isomerase, **E**: Caffeoyl-CoA O-methyltransferase, **F**: L-ascorbate peroxidase, **G**: Probable glutathione S-transferase GSTF1, **H**: Superoxide dismutase [Mn] 3.1, **I**: Isoflavone reductase homolog, **J**: Auxin-induced protein PCNT115, **K**: 14-3-3-like protein GF14-6, and **L**: Late embryogenesis abundant protein. Each bar represents the mean values ± standard deviation for three biology replicates.

In order to verify the changed expression of mRNA from three banana cultivars in response to Foc4 infection and evaluate the correlation of transcriptional and protein level, twelve randomly genes involved in defense, secondary metabolism, oxidative-redox stress, signal conduction, molecular chaperones and primary metabolism were selected to validate through qRT-PCR method. In our study, the transcript levels of the pathogenesis-related protein 1, protein IN2-1 homolog B, L-ascorbate peroxidase, probable glutathione S-transferase GSTF1, isoflavone reductase homolog, auxin-induced gene PCNT115 and 14-3-3-like gene GF14-6 were well consistent with their protein levels after pathogen infection, while the levels of five transcripts did not correspond with their protein levels (Figure 6). Because gene expression is controlled at multiple regulatory levels at their transcriptional, post-transcriptional, translational, and post-translational levels, the validity of the correlation of mRNA and protein expression levels requires further study.

## Discussion

The defense mechanism of other crops in response to infection has been widely studied at the molecular level. However, the defense mechanism of banana in response to infections remains obscure. To investigate dynamic changes in induced proteins, proteome profile analyses of three different banana roots were successfully performed. The significant differences of defense levels in banana roots were displayed at the time-points of 3D after Foc4 or H_2_O treatment. The defense proteins acting in response to *F. oxysporum* infection are involved in complex and effective pathways from susceptible ‘Brazil’ to highly resistant ‘Yueyoukang I’. A total of 43 differentially expressed proteins with known functions were identified by MALDI-TOF/TOF analysis; they were subsequently classified into nine categories according to their functions. These induced proteins play important roles in defense as a response to pathogen infection. The elucidation of these proteins is beneficial in understanding the complex resistance mechanisms against the fungal pathogen *F. oxysporum* at the proteome level.

### Defense response proteins

PR proteins are effective in responding to fungal, bacterial, and viral infections. Consequently, this response is related to the development of systemic acquired resistance (SAR). Proteome analyses revealed that two PR proteins involved in defense were induced in both resistant and susceptible banana after pathogen infection. The proteins were identified as pathogenesis-related protein 1 (PR-1; No. 2) and RecName: Full = alpha-amylase inhibitor/endochitinase (No. 16). PR-1 is a dominant defense protein induced by pathogens, SA, or ET, which is commonly considered as a marker for SAR [27]. The expression patterns of PR-1 in response to oomycete and fungal pathogens were positively regulated in many plants [28]. For example, the antifungal protein of PR-1 in response to *F. oxysporum* was up-regulated in both GCTCV-218 and Williams [21]. RecName: Full = alpha-amylase inhibitor/endochitinase is a protein purified from the seeds of Job's Tears (*C. lachryma-jobi* L.); the antifungal protein was up-regulated to protect plants from insect feeding and fungal infection [29]. Two defense proteins were up-regulated in banana, of which the proteins PR-1 and RecName: Full = Alpha-amylase inhibitor/endochitinase were differentially regulated in susceptible ‘Brazil’ and highly resistant ‘Yueyoukang I’. Interestingly, PR-1 protein was only found in moderately resistant ‘Nongke No.1’. The results indicated that more defense genes were expressed in response to fungal infection in highly resistant ‘Yueyoukang I’ and susceptible ‘Brazil’ than moderately resistant ‘Nongke No.1’ , where other plant defense pathways were regulated in moderately resistant ‘Nongke No.1’.

### Secondary metabolism-related proteins

Some enzymes associated with secondary metabolism play key roles in the synthesis of antifungal compounds and cell wall components, which are involved in the phenylpropanoid pathway and other pathways. In our study, four proteins involved in the phenylpropanoid pathway were identified as caffeoyl-CoA O-methyltransferase (CCOMT; No. 28), isoflavone reductase homolog (so-called IFR “homolog”; No. 32) and leucoanthocyanidin dioxygenase (LDOX; No. 34). S-adenosylmethionine synthase (SAM; No. 36) was identified as another protein in the secondary metabolism pathway. The proteins in the phenylpropanoid pathway were classified into three sub-pathways based on the following criteria: (i) isoflavonoids, (ii) flavonoids and anthocyanins, and (iii) lignin biosynthesis [30]. The IFR “homolog” is highly similar to IFR in catalyzing the synthesis of isoflavonoid phytoalexin medicarpin. IFR in alfalfa was rapidly induced in response to fungal pathogen [31]. The protein was up-regulated in highly resistant ‘Yueyoukang I’ and moderately resistant ‘Nongke No.1’. The results indicated that a large number of phytoalexins were synthesized to protect banana from Foc4 infection. CCOMT is a branch point enzyme of monolignol biosynthesis, which is beneficial for the lignifications of the cell wall in response to pathogen infection [32]. Down-regulation of CCOMT in transgenic alfalfa reduces guaiacyl lignin biosynthesis [33]. This protein was up-regulated in moderately resistant ‘Nongke No.1’. The results implied that the lignifications of the cell wall have important functions in response to Foc4 infection. LDOX is a key regulated enzyme for anthocyanin synthesis, which was up-regulated in response to various abiotic and biotic stresses, such as pathogen infection, UV light, high-intensity light, temperature, wounding, drought, and nutrient deficiency [34,35]. The protein was observed to have disappeared in moderately resistant ‘Nongke No.1’ , which decreased end-products at the later stage of the pathway. Furthermore, the enzymes of the flavonoid pathway were not detected to change significantly in moderately resistant ‘Nongke No.1’. Consistent with our study, the defense of moderately resistant ‘Nongke No.1’ to Foc4 apparently does not dependent on the anthocyanin and flavonoid pathway. Overall, the different changes in the proteins of the anthocyanin and flavonoid pathways indicated that the plant might be transferring substrates between the flavonoid and anthocyanin pathways to increase the production of lignin and isoflavonoids. SAM indirectly plays an important role in plant defense by catalyzing the production of S-adenosyl-L-methionine, which supplies as a methyl-group donor in the biosynthesis of numerous secondary metabolites [36]. This protein can increase plant resistance in response to various abiotic stresses [37-39]. SAM was observed to have disappeared in moderately resistant ‘Nongke No.1’. These results indicated that substrates of other secondary metabolism pathways were transferred into the synthesis of lignin and isoflavonoids in response to Foc4 infection.

### Polysaccharose synthesis

Protein No. 42 was identified as alpha-1, 4-glucan-protein synthase (UDP-forming). UDP-forming is associated with the formation of cell wall polysaccharoses, including hemicellulosic and xylose, which is beneficial for the formation of physical barriers [40]. UDP-forming was up-regulated in maize in response to *Trichoderma harzianum* T22 [41]. Although the deposition of polysaccharoses in the cell wall confined the fungi to the outer root layer, the expression of UDP-forming was only up-regulated in highly resistant ‘Yueyoukang I’. Such results indicated that the formation of complex cell wall with different cell components is one of most effective defense strategies in response mechanisms to fungal degradation. This strategy was more evidently observed in highly resistant ‘Yueyoukang I’ than moderately resistant ‘Nongke No.1’ and susceptible ‘Brazil’.

### Cell cytoskeletion

Protein No. 37 was identified as actin, which constitutes cell cytoskeleton that is involved in cell polarization defense in response to microbial attack. During the early stages of fungal infection, the activity of the actin pathway plays an important role in cell polarization defense by trafficking and secreting antimicrobial compounds to the infection site and sediment barrier material to the penetrated sites for cell wall enhancement, accompanied by the activity of defense gene PR-1 [42-44]. By contrast, in the present study, the protein was only up-regulated in moderately resistant ‘Nongke No.1’. This result indicated that cell polarization defense may be more effective for early fungal infection in moderately resistant ‘Nongke No.1’ than in highly resistant ‘Yueyoukang I’ and susceptible ‘Brazil’.

### Oxidative-redox stress

The rapid accumulation of ROS with their toxic potential, a phenomenon called oxidative burst, is a nearly ubiquitous response to abiotic and biotic stresses in plants. In the present study, only one protein was identified as Germin-like protein 12–1 (GLP, spot Nos. 11, 12, 13 and 15), which produces ROS. However, the high levels of ROS can lead to severe oxidative destruction of cell structures, such as nucleic acids, proteins, and lipids. To regulate oxidative-redox homeostasis, four antioxidant proteins were identified as protein IN2-1 (IN2-1; protein No. 14), L-ascorbate peroxidase (APX; protein No.29), glutathione S-transferase GSTF1 (GST; protein No. 30), and superoxide dismutase [Mn] (SOD; protein No. 31), which were activated in antioxidant systems to scavenge ROS. GLP catalyzes the production of H_2_O_2_ as oxalate oxidase or SOD in plant defense, which is induced by various stresses [45]. SOD (EC 1.15.1.1) is believed to be among the first line of defense proteins to scavenge ROS, which catalyzes the dismutation of superoxide to H_2_O_2_ and oxygen [46]. The protein is also related to the activation of APX involved in the pathways of ascorbate and glutathione [47]. APX is a key enzyme that converts H_2_O_2_ to water, in addition to regulating redox signal transduction [48]. Protein IN2-1 is identified as a homolog of GST. In addition to their functions in herbicide detoxification, stress signaling conduction, and apoptosis regulation, GST and protein IN2-1 are glutathione peroxidases that function in ROS scavenging in various stresses [49]. The rapid accumulation of GLP is beneficial for resistance against rice blast and sheath blight [50]. The expression patterns of IN2-1/GST are complex under pathogen infection. For example, the infection caused by *Aphanomyces euteiches* (oomycota) increases the synthesis of IN2-1/GST in infected *Medicago truncatula*[51]. By contrast, the infection caused by rust fungi decreases their synthesis in infected bean [52]. GLP were up-regulated in three banana cultivars. Meanwhile, IN2-1 was up-regulated in susceptible ‘Brazil’ and moderately resistant ‘Nongke No.1’. These results indicated that maintenance of high levels of ROS is beneficial to defense effectively against the pathogen infection. In our study, the antioxidant enzymes APX, GST, and SOD were down-regulated in moderately resistant ‘Nongke No.1’. This finding is contrary to the results of up-regulated proteins in response to *F. oxysporum*[22]. These results indicated that antioxidative emzyme IN2-1 were induced to defense effectively against the pathogen infection in moderately resistant ‘Nongke No.1’ than the antioxidant enzymes APX, GST, and SOD.

### Signal conduction

Host defense mechanisms were triggered by various early signal pathways in response to pathogen infection. Proteomic analysis showed that the signal proteins related to JA, ABA, and auxin in signaling pathways were differentially regulated in banana. Six spots were identified as signal proteins: putative horcolin (No. 1), abscisic stress-ripening protein 3 (ASR3; No. 8), abscisic stress ripening protein (ASR, No. 33), auxin-induced protein PCNT115 (AIP; No. 35), 14-3-3-like protein GF14-E (No. 38), and 14-3-3-like protein GF14-6 (No. 39). Horcolin is a new jacalin-related lectin specific to mannose, which may play an important role in the perception and transduction of hormones in response to abiotic/biotic stress [53]. The expression of TaJRLL1, a jacalin-related lectin in transgenic *A. thaliana*, increases resistance to *Fusarium graminearum* and *Botrytis cinerea*, which may be involved in the SA- and JA-dependent defense signal pathways [54]. ASR3 and ASR are stress-inducible proteins involved in ABA signal pathways, which respond to various stresses, including pathogen, drought, salt, cold, osmotic pressure, ABA, and injury [55]. ASR was up-regulated in soybean root in response to *Fusarium solani* f. sp. *glycines*[56]. Horcolin was induced in susceptible ‘Brazil’. On the contrary, ARS3 were down-regulated in susceptible ‘Brazil’. These results indicated that defense was regulated together by the conduction of JA and ABA signals. ARS was up-regulated in moderately resistant ‘Nongke No.1’ , signifying the active defense by the conduction of ABA signal. AIP is related to plant defense and auxin-mediated plant growth. Auxin and defense-related antimicrobial secondary metabolites are produced through the same tryptophan pathway [57]. The AIP in grapevine was down-regulated in response to phytoplasma infection [58]. In accordance with these previous results, the present results showed that AIP was down-regulated in moderately resistant ‘Nongke No.1’. This result indicated that the substrates of the auxin pathway were transferred into the production of defense-related secondary metabolites. The 14-3-3 family comprises ubiquitous proteins, which play important roles as regulators in plant cells; specifically, these proteins function in nitrogen assimilation, sucrose synthesis, H^+^-pump activation, and ABA signal transduction [59]. Moreover, 14-3-3-like proteins in barley were up-regulated in response to powdery mildew fungus [60]. Accordingly, the present results showed that the 14-3-3-like protein GF14-E and 14-3-3-like protein GF14-6 were up-regulated in highly resistant ‘Yueyoukang I’. This result indicated that various plant defenses were induced upon pathogen infection.

### Molecular chaperones

Proteins function as molecular chaperones by maintaining cellular homeostasis, which includes protein folding, assembly, translocation, and degradation; protein and membrane stabilization; and refolding of proteins to nonnative state under stressful conditions [61]. In the present study, four proteins were identified as molecular chaperones, namely, heat shock 22 kDa protein (HSP 22; protein Nos. 9 and 10), T-complex protein 1 subunit gamma (TCP-1; protein No.22), protein disulfide-isomerase (PDI; protein No. 26), and late embryogenesis abundant protein (LEA; protein No. 40). HSP 22, a mitochondrial protein, is identified as a molecular chaperone that functions in facilitating the reactivation of denatured proteins and preventing the aggregation of heat-induced proteins [62]. TCP-1 aids in the folding and reassembly of some proteins, including nucleotide-binding proteins, actin, tubulin, and luciferase [63]. PDI acts as a molecular chaperone from the ferredoxin family, whose biochemical function in plant mitochondria remains unclear. However, reductive PDI has been associated to various roles, including removal of aberrant disulfides, folding of new proteins, and reduction of disulfides required in activating proteins involved in antioxidant defense [64]. LEA is a hydrophilic protein that acts as a molecular chaperone to maintain the stabilization of proteins, vesicles, and endomembranes in the sequestration of ions by binding water molecules [65]. In susceptible ‘Brazil’, HSP 22 was up-regulated, whereas TCP-1 and PDI were down-regulated. LEA was up-regulated in highly resistant ‘Yueyoukang I’. These results indicated that the proteins had different roles as molecular chaperones in the defense response to pathogen infection.

### Energy metabolism

Protein No. 21 was identified as ferredoxin-NADP reductase. This protein catalyzes the production of NADPH to be supplied in CO_2_ fixation; however, a substantial fraction of NADPH is involved in the electron transport of various cell pathways, such as N and S assimilation and amino acid and fatty acid metabolism [66]. Aside from maintaining the homeostasis of NADPH/NADP, the protein is also considered as a key antioxidant that scavenges ROS under various environmental stresses [67]. The protein was up-regulated in susceptible ‘Brazil’ , suggesting that the protein is necessary to supply energy for the metabolic changes in infected plant cells and to reduce the damage of ROS.

### Primary metabolism

Six proteins were involved in primary metabolism: glutamine synthetase nodule isozyme (GS; Nos. 17, 18 and 19), putative carboxyvinyl-carboxyphosphonate phosphorylmutase (CPPM; No. 20), NADP-dependent malic enzyme (NADP-ME; No. 23), putative aconitate hydratase (Nos. 24 and 25), fructokinase-1 (FRK-1; protein No. 27), fructokinase-2 (FRK-2; No. 41), and malate dehydrogenase (MDH; No. 43).

NADP-ME is an ubiquitous enzyme catalyzing malate for the production of pyruvate and NADPH, which could subsequently provide NADPH for the syntheses of lignin, flavonoid, and NADPH oxidase in plant defense [68,69]. NADP-ME expression is up-regulated in defense responses in transgenic tobacco [70]. It is rapidly induced in the defense response to *Magnaporthe grisea*[71]. The protein was down-regulated in susceptible ‘Brazil’. Such results indicated that the suppression of lignin, flavonoid, and NADPH oxidase synthesis in susceptible banana causes a weak defense in response to Foc4 infection.

GS is a key enzyme catalyzing the ammonia assimilation and phenyl propanoid pathway in plants, which confers environment stress tolerance [72]. GS was down-regulated in susceptible ‘Brazil’, indicating that the synthesis of defense compounds was increased in defense reactions. CPPM mainly catalyzes the formation of an unusual C-P bond in the processing of metabolites. To date, data linking this role in plants are lacking; however, the protein is well known for the synthesis of antibiotic bialaphos in microorganisms [73]. CPPM was up-regulated in susceptible ‘Brazil’. The roles of these proteins need to be further investigated in the future. Aconitate hydratase and MDH are key enzymes for citric acid metabolism, also known as the tricarboxylic acid cycle (TCA cycle). The protein of aconitate hydratase catalyzes the production of citrate or isocitrate [74]. MDH is also an important enzyme involved in multiple metabolic pathways of TCA and photosynthesis, catalyzing the reversible reduction of oxaloacetate to malate [75]. Aconitate hydratase and MDH were down-regulated in susceptible ‘Brazil’ and highly resistant ‘Yueyoukang I’ , respectively. These results indicated that energy and substrates exhausted in metabolism were supplied by the other pathways. FRK-1 and FRK-2 are key enzymes of glycolysis in plants, which are both involved in the stress response of plant cells. Both enzymes also catalyze the phosphorylation of fructose to fructose-6-phosphate, which is a major substrate for many sugar metabolic pathways involved in glycolysis, starch biosynthesis, and the oxidative pentose pathway [76,77]. In addition, fructokinases important functions as sugar sensors or signals in plant defense [78]. FRK-1 was up-regulated in susceptible ‘Brazil’ , whereas FRK-1 and FRK-2 were up-regulated in highly resistant ‘Yueyoukang I’. These results indicated that the conduction of sugar signal can significantly strengthen plant defense.

## Conclusions

This study is the first to perform protein profiling of banana roots in response to Foc4 infection. Proteome analyses revealed that various proteins are involved in complex defense pathways, including PR-related proteins, secondary metabolites, signal conduction proteins, cell wall polysaccharose synthesis proteins, cell polarization defense proteins, and oxidative-redox homeostasis proteins. The temporal expression patterns of pathogen-responsive proteins in resistant and susceptible banana cultivars may provide useful information about the defense mechanisms of banana. These mechanisms were more evident in moderately resistant ‘Nongke No.1’ and highly resistant ‘Yueyoukang I’ than in susceptible ‘Brazil’. The proteins of PR and antifungal compounds were found to play main roles in the chemical resistance of banana cultivars against the fungal infection in the experiment. PR proteins were induced in three banana cultivars. Antifungal compound synthesis proteins were induced in moderately resistant ‘Nongke No.1’ and highly resistant ‘Yueyoukang I’. Furthermore, other secondary metabolites were only rapidly induced in moderately resistant ‘Nongke No.1’ , which was considered to be a more effective pathway in moderately resistant ‘Nongke No.1’ than in highly resistant ‘Yueyoukang I’. Meanwhile, the structure of physical barriers was believed to confine the expansion of Foc4 in extracellular tissues based on cell wall thickness and lignifications, and polysaccharide precipitation. The lignin synthesis-related protein was down-regulated and up-regulated in susceptible ‘Brazil’ and moderately resistant ‘Nongke No.1’ , respectively. Moreover, the cell wall polysaccharide synthesis-related protein was induced in highly resistant ‘Yueyoukang I’. Finally, the defense mechanism is coordinated by several enzymes, such as signal-conductor proteins and molecular chaperones. Above all, PR and ROS were found to play main roles in defense in susceptible ‘Brazil’. PR, ROS and antifungal compounds at the infected sites and cell wall lignifications play roles in defense in moderately resistant ‘Nongke No.1’. Meanwhile, PR, antifungal compounds, and complex cell wall proteins play roles in defense in highly resistant ‘Yueyoukang I’. 12 randomly proteins involved in defense, secondary metabolism, oxidative-redox stress, signal conduction, molecular chaperones and primary metabolism were performed to validate the correlation of transcriptional and protein levels through qRT-PCR. Transcriptional levels of most selected genes were consistent with their respective protein abundance after pathogen infection. The present study provided important clues for understanding the defense mechanism of banana against pathogen infection. Our results will be useful in designing strategies to cure and control diseases.

## Methods

### Plant materials and Foc 4 inoculation

Three banana cultivars belonging to the same genomic group were selected in this study, including banana cv. ‘Brazil’ (*Musa acuminata* L.; AAA Group cv. Brazil), banana cv. ‘Nongke No.1’ (*M. acuminata* L.; AAA Group cv. Nongke No.1), and banana cv. ‘Yueyoukang I’ (*M. acuminata* L.; AAA Group cv. Yueyoukang I). ‘Brazil’ is susceptible to *F. oxysporum*, whereas ‘Nongke No.1’ and ‘Yueyoukang I’ are moderately and highly resistant to this pathogen, respectively. Tropical Race 4 strain of *F. oxysporum* f. sp*. cubense* (Foc4) has been under long-term preservation in our laboratory. All cultivars were tissue cultured as described by Ganapathi et al. [79]. Banana plantlets were inoculated using the method of Berg et al. [20] with some modifications. Propagated banana plantlets were transferred into 85 mm diameter tissue boxes containing sterile peat soil. The plantlets were incubated in a greenhouse at 21°C to 28°C with a photoperiod of 12 h:12 h light:dark. When the plantlets reached the four-leaf stage, washed roots of the plantlets were wounded by gentle crushing prior to inoculation with the pathogen. Then, roots and rhizomes were dipped into a 500 mL sterile beaker containing 200 mL tropical race 4 spore suspension (10^6^ conidia/mL of each isolate) and then incubated for 30 min. The control plants were treated with sterile distilled water in the trial. Each sample set-up was done in triplicates. Each treated plant was subsequently planted singly in a 200 mL plastic cup with steam-sterilized soil, placed on iron trays, and then kept in a humidity chamber in a greenhouse (28 ± 1°C and a natural photoperiod of 12 h:12 h light/dark). Root samples were collected at the point of 3D after Foc4 or H_2_O treatment. The samples were flash frozen in liquid nitrogen and stored at −80°C.

### Protein extraction

Proteins were extracted and separated from the samples according to the protocol of Isaacson et al. [80]. Briefly, mortar and pestle were chilled with liquid nitrogen before grinding, and 1 g of root tissue was frozen in liquid nitrogen. Samples were ground to fine power with a pestle in liquid nitrogen and then suspended in 10 mL extraction buffer (0.7 M sucrose, 0.1 M KCl, 0.5 M Tris–HCl, 50 mM EDTA and 0.2% DTT, pH 7.5). Total proteins were precipitated overnight at −20°C. Subsequently, the mixtures were centrifuged at 5,000 g for 30 min at 4°C. The sediment pellets were washed three or five times with 10 mL of ice-cold methanol containing 0.2% DTT. The collected pellets were air-dried at 4°C and then re-suspended in lysis buffer [7 M urea, 2 M thiourea, 1% CHAPS, 2% ampholytes (pH 4 to 7), 1% DTT]. Finally, the mixtures were centrifuged at 16,000 g for 10 min at 4°C, and protein supernatants were transferred to new tubes. The protein concentrations were estimated using the Bradford assay [81].

### 2-DE and gel staining

The lysed samples were diluted with rehydration buffer (7 M urea, 2 M thiourea, 4% CHAPS, 0.002% bromophenol blue, 0.5% IPG buffer, 75 mM DTT) to 380 μL protein supernatant containing 550 μg protein. Subsequently, 18 cm IPG strips (pI 4 to 7, GE Healthcare) were rehydrated for at least 12 h in diluted protein. Ettan IPGphor II (GE Healthcare) was used to perform IEF at 20°C with a current limit of 50 mA per strip. The program was set as follows: 12 h at 50 V, 2 h at 500 V, 2 h at 1000 V, 7 h at 8000 V (gradient), and 50000 Vh at 8000 V. After isoelectrical focus of proteins was completed, each strip was individually equilibrated for 15 min in 8 mL equilibration solution (6 M urea, 30% glycerol, 2% SDS, 0.002% bromophenol blue, 50 mM Tris, pH 8.8) containing 1% DTT and then for 15 min in 8 mL equilibration buffer containing 2.5% iodoacetamide. Second-dimension separation was carried out on an Ettan DALT 6 System (GE Healthcare) using 12% SDS polyacrylamide gels. Protein spots of gels were visualized by CBB G-250 staining [82]. Three replicate gels were run for each sample.

### Image analysis

The stained gels were scanned with ImageScanner (GE Healthcare), and the images were statistically analyzed using PDQuest software (Version 8.0, Bio-Rad, Hercules, CA, USA). The protein expression profiles of control tissues were used as a reference. Then, the matched spots were analyzed manually, and falsely matched or unmatched spots were corrected. Percentage spot volumes were calculated using Student’s t-test, and spots with twofold abundance changes were selected as significant differential proteins.

### Protein digestion

The differential protein spots were excised from the gel. Tryptic digestion of the selected spots was carried out as described by Guo et al. [83] with some modifications. Briefly, each excised spot was de-stained with 100 mM NH_4_HCO_3_ in 30% ACN. The gel pieces were minced, lyophilized, and then rehydrated in trypsin containing NH_4_HCO_3_ at 37°C overnight. After digestion, the peptides were collected, and the pellets were washed with 0.1% TFA in 60% (v/v) ACN three times to dry the remaining peptides.

### MALDI-TOF/TOF MS analysis and database searching

The differential proteins were identified by MALDI-TOF-TOF as described previously [84]. The MS and MS/MS spectra were acquired using an ABI 4700 Proteomics Analyzer (Applied Biosystems, Foster City, CA, USA). Data search files were generated by GPS-Explorer Software 3.6 (Applied Biosystems), and then sequence similarity searches in the *M. acuminate* protein database (http://banana-genome.cirad.fr/content/download-dh-pahang), MASCOT NCBInr database (http://www.matrixscience.com/cgi/search_form.pl?FORMVER=2&SEARCH=SQ) and Plant_EST database were performed with MultiAgent Supply Chain cOordination Tool (MASCOT) search engine 2.2 (Matrix Science, United Kingdom) with default parameters based on peptide mass fingerprinting PMF and MS/MS spectra.

### Quantitative RT-PCR analysis

RNA samples were extracted from banana roots using E.Z.N.A.TM plant RNA mini Kit (Omega, USA). 0.5 μg total RNA as template was reverse transcription as first-strand cDNA using iscriptTM advanced cDNA Synthesis Kit (Bio-Rad) according to the manufacturer’s instructions. Quantitative real time PCR was performed using the ssoFastTM evagreen supermix(Bio-Rad) on a Takara Thermal Cycler Device for Real-Time System(Takara). Primers listed in Table 1 are designed based on *M. acuminate* database. And α-tublin have been traditionally used as the endogenous reference gene [85]. 20 μl PCR reactions consisted of 10 μl ssoFast evagreen supermix, 2 μl template (10 × diluted cDNA), and 20μm of each primer (forward and reverse). Each reaction was incubated in PCR Strip Tubes at 95°C for 30 s, followed by 40 cycles of 95°C for 5 s, 58°C for 30 s. Three independent biological replicates were run for each sample. For each biological replicate, three technical replicates were used for qRT-PCR analyses.

**Table 1:** Primer sequences used for qRT-PCR

**Spot no.**	**Protein name**	**Forward primer (5’ → 3’)**	**Reverse primer (5’ → 3’)**	**Size (bp)**
2	Pathogenesis-related protein 1	TTGGGTACGTGAAGGAAAGA	GGTAGGCTGATGGGTTGG	92
14	Protein IN2-1 homolog B	CTTTCTTCCTTGGGCAATTCAG	TCTGGAACCTCTCAACAAATGGA	66
23	NADP-dependent malic enzyme	GCACATATTGCCGCTAACGTT	CAGCCGGGTTGCCAAAC	60
26	Protein disulfide-isomerase	CCGCATCGGTGTTGAGTAAA	ACATCTTCGTTTGCATCAACCTT	67
28	Caffeoyl-CoA O-methyltransferase	GATCAACGCCAAGAACACCAT	GGTGGCGAGGAGGGAGTAC	61
29	L-ascorbate peroxidase	GTGGACACACACTGGGAAGGT	AGTCCAAGCCCCCTCAAAA	58
30	Probable glutathione S-transferase GSTF1	GCGATTTCCGCCATCTACAT	ACGCCGCCGAAGAAGAC	57
31	Superoxide dismutase [Mn] 3.1	TGGATAAGGGAAGTAAGAAAC	TGCCCAGCAAAGGAAC	93
32	Isoflavone reductase homolog	CCAAGAGGCGCCAATCC	CAAAGGCGGAGTGGCAAATA	56
35	Auxin-induced protein PCNT115	AAGGCGTGCGCATGCT	CCACAGCGACCACTCCATCT	58
39	14-3-3-like protein GF14-6	GGATATTGCCCTGGCTGAAC	GTGCCAGCCCCAACCTAAT	56
40	Late embryogenesis abundant protein	TCGTGGATGTGCCAATTTTC	GGGATCTCGCCAGTCTTCTCT	61
	Tubulin	TGTTGCATCCTGGTACTGCT	GGCTTTCTTGCACTGGTACAC	112

## Abbreviations

Foc4: *Fusarium oxysporum* f. sp. *cubense* tropical race 4; PR: Pathogenesis-related; F. oxysporum: *Fusarium oxysporum* f. sp. *cubense* (E.F. Smith) Snyder and Hansen; PAMP: Pathogen-associated molecular pattern; PTI: Pathogen-associated molecular pattern (PAMP)-triggered immunity; PRRs: Pattern-recognition Receptors; ROS: Reactive oxygen species; ETI: Effector-triggered immunity; SA: Salicylic acid; JA: Jasmonic acid; ET: Ethylene; ABA: Abscisic acid; 2-DE: Two-dimensional electrophoresis; PI: Isoelectric point; CBB: Coomassie Brilliant Blue; MALDI-TOF/TOF: Matrix-assisted laser desorption/ionization time-of-flight/time-of-flight; QRT-PCR: Quantitative real time PCR method; ESTs: Expressed sequence tags; SAR: Systemic acquired resistance; CCOMT: Caffeoyl-CoA O-methyltransferase; IFR: Isoflavone reductase; LDOX: Leucoanthocyanidin dioxygenases; SAM: S-adenosylmethionine synthase; UDP-forming: Alpha-1, 4-glucan-protein synthase; GLP: Germin-like protein 12–1; IN2-1: Protein IN2-1; APX: L-ascorbate peroxidase; GST: Glutathione S-transferase GSTF1; SOD: Superoxide dismutase [Mn]; ASR3: Abscisic stress-ripening protein 3; ASR: Abscisic stress ripening protein; AIP: Auxin-induced protein PCNT115; HSP 22: Heat Shock 22 kDa protein; TCP-1: T-complex protein 1 subunit gamma; PDI: Protein disulfide-isomerase; LEA: Late embryogenesis abundant protein; GS: Glutamine synthetase nodule isozyme; CPPM: Putative carboxyvinyl-carboxyphosphonate phosphorylmutase; NADP-ME: NADP-dependent malic enzyme; FRK-1: Fructokinase-1; FRK-2: Fructokinase-2; MDH: Malate dehydrogenase; TCA: Tricarboxylic acid; MOSCOT: MultiAgent Supply Chain cOordination Tool.

## Competing interests

We declare that no competing interests exist.

## Authors' contributions

Conceived and designed the experiments: XL and HL. Performed the experiments: XL and YL Analyzed the data: XL, YL and HL. Contributed reagents/materials/analysis tools: TB and XR. Wrote the paper: XL, TB and HL. All authors read and approved the final manuscript.

## Supplementary Material

Additional file 1: Table S1 Identification of differentially expressed proteins from banana root inoculated with Foc4 by MALDI-TOF/TOF MS. Click here for file

## References

[B1] Wibowo A Subandiyah S Sumardiyono C Sulistyowati L Taylor PMF Occurrence of tropical race 4 of *fusarium oxysporum* f. sp. *cubense* in indonesia Plant Pathol 2011 27 280 284

[B2] Saravanan T Muthusamy M Marimuthu T Development of integrated approach to manage the fusarial wilt of banana Crop Prot 2003 22 1117 1123

[B3] Hwang SC Ko WH Cavendish banana cultivars resistant to fusarium wilt acquired through somaclonal variation in taiwan Plant Dis 2004 88 581 588 10.1094/PDIS.2004.88.6.580 30812575

[B4] Dital MA Waalwijk C Buddenhagen IW Souza MT Jr Kema GHJ A molecular diagnostic for tropical race 4 of the banana fusarium wilt pathogen Plant Pathol 2010 59 348 357

[B5] Hennessy C Walduck G Daly A Padovan AC Weed hosts of *Fusarium oxysporum* f. sp *cubense* tropical race 4 in northern Australia Australasian Plant Pathol 2005 34 115 117

[B6] Michielse CB Rep M Pathogen profile update: *Fusarium oxysporum* Mol Plant Pathol 2009 10 311 324 19400835 10.1111/j.1364-3703.2009.00538.x PMC6640313

[B7] Li CY Chen S Zuo CW Sun QM Ye Q Yi GJ Huang BZ The use of GFP-transformed isolates to study infection of banana with *Fusarium oxysporum* f. sp. *cubense* race 4 Eur J Plant Pathol 2011 131 327 340

[B8] Baysal O Lai D Xu HH Siragusa M Mikail C Carimi F Da Silva JAT Tor M A Proteomic Approach Provides New Insights into the Control of Soil-Borne Plant Pathogens by *Bacillus* Species PLOS ONE 2013 1 e53182 23301041 10.1371/journal.pone.0053182 PMC3536778

[B9] Tsuda K Katagiri F Comparing signaling mechanisms engaged in pattern-triggered and effector-triggered immunity Curr Opin Plant Biol 2010 13 459 465 20471306 10.1016/j.pbi.2010.04.006

[B10] Schwessinger B Zipfel C News from the frontline: recent insights into PAMP-triggered immunity in plants Curr Opin Plant Biol 2008 11 389 395 18602859 10.1016/j.pbi.2008.06.001

[B11] Chisholm ST Coaker G Day B Staskawicz BJ Host-microbe interactions: shaping the evolution of the plant immune response Cell 2006 124 803 814 16497589 10.1016/j.cell.2006.02.008

[B12] He P Shan LB Sheen J Elicitation and suppression of microbe-associated molecular pattern-triggered immunity in plant-microbe interactions Cell Microbiol 2007 9 1385 1396 17451411 10.1111/j.1462-5822.2007.00944.x

[B13] Zhang J Lu HB Li XY Li Y Cui HT Wen CK Tang XY Su Z JM Z Effector-triggered and pathogen-associated molecular pattern–triggered immunity differentially contribute to basal resistance to *Pseudomonas syringae* Mol Plant Microbe In 2010 23 940 948 10.1094/MPMI-23-7-0940 20521956

[B14] Nomuraa K Meceya C Lee YN Imboden LA Changc JH He SY Effector-triggered immunity blocks pathogen degradation of an immunity-associated vesicle traffic regulator in Arabidopsis Proc Natl Acad Sci USA 2011 108 10774 10779 21670267 10.1073/pnas.1103338108 PMC3127868

[B15] Dodds PN Rathjen JP Plant immunity: towards an integrated view of plant-pathogen interactions Nat Rev Genet 2010 11 539 548 20585331 10.1038/nrg2812

[B16] Bari R Jones JDG Role of plant hormones in plant defence responses Plant Mol Biol 2009 69 473 488 19083153 10.1007/s11103-008-9435-0

[B17] Zarate SI Kempema LA Walling LL Silverleaf whitefly induces salicylic acid defenses and suppresses effectual jasmonic acid defenses Plant Physiol 2007 143 866 875 17189328 10.1104/pp.106.090035 PMC1803729

[B18] Mauch-Mani B Mauch F The role of abscisic acid in plant-pathogen interactions Curr Opin Plant Biol 2005 8 409 414 15939661 10.1016/j.pbi.2005.05.015

[B19] Kazan K Manners JM Linking development to defense: auxin in plant-pathogen interactions Trends Plant Sci 2009 14 1360 1385 10.1016/j.tplants.2009.04.005 19559643

[B20] Singer AC Crowley DE Thompson IP Secondary plant metabolites in phytoremediation and biotransformation Trends Biotechnol 2003 21 123 130 12628369 10.1016/S0167-7799(02)00041-0

[B21] Van Den Berg N Berger DKHI Birch PRJ Wingfield MJ Viljoen A Tolerance in banana to Fusarium wilt is associated with early up-regulation of cell wall-strengthening genes in the roots Mol Plant Pathol 2007 8 333 341 20507503 10.1111/j.1364-3703.2007.00389.x

[B22] Li CY Deng GM Yang J Viljoen A Jin Y Kuang RB Zuo CW Lv ZC Yang QS Sheng O Transcriptome profiling of resistant and susceptible Cavendish banana roots following inoculation with *Fusarium oxysporum* f. sp. *cubense* tropical race 4 BMC Genomics 2012 13 374 22863187 10.1186/1471-2164-13-374 PMC3473311

[B23] DHont A Denoeud F Aury JM Baurens FC Carreel F Garsmeur O Noel B Bocs S Droc G Rouard M The banana (*Musa acuminata*) genome and the evolution of monocotyledonous plants Nature 2012 488 213 217 22801500 10.1038/nature11241

[B24] Carpentier SC Witters E Laukens K Van Onckelen H Swennen R Panis B Banana (*Musa spp.*) as a model to study the meristem proteome: acclimation to osmotic stress Proteomics 2007 7 92 105 17149779 10.1002/pmic.200600533

[B25] Yang QS Wu JH Li CY Wei YR Sheng O Hu CH Kuang RB Huang YH Peng XX McCardle JA Quantitative proteomic analysis reveals that antioxidation mechanisms contribute to cold tolerance in plantain (*Musa paradisiaca* L.; ABB Group) seedlings Mol Cell Proteomics 2012 11 853 869 10.1074/mcp.M112.022079 PMC3518116 22982374

[B26] Soares NC Cabral MP Parreira JR Gayoso C Barba MJ Bou G 2-DE analysis indicates that *Acinetobacter baumannii* displays a robust and versatile metabolism Proc Natl Acad Sci U S A 2009 7 37 10.1186/1477-5956-7-37 PMC2761859 19785748

[B27] Sarowar S Kim YJ Kim EN Kim KD Hwang BK Islam R Shin JS Overexpression of a pepper basic pathogenesis-related protein 1 gene in tobacco plants enhances resistance to heavy metal and pathogen stresses Plant Cell Rep 2005 24 216 224 15719238 10.1007/s00299-005-0928-x

[B28] Van Loon L Strien E The families of pathogenesis-related proteins, their activities, and comparative analysis of PR-1 type proteins Physiol Mol Plant P 1999 55 85 97

[B29] Ary MB Richardson M Shewry PR Purification and characterization of an insect alpha-amylase inhibitor endochitinase from seeds of job tears (*Coix lachryma-jobi*) Biochim Biophys Acta 1989 999 260 266 2605263 10.1016/0167-4838(89)90007-1

[B30] Zabala G Zou J Tuteja J Gonzalez DO Clough SJ Vodkin VO Transcriptome changes in the phenylpropanoid pathway of Glycine max in response to *Pseudomonas syringae* infection BMC Plant Biol 2006 6 26 17083738 10.1186/1471-2229-6-26 PMC1636052

[B31] Paiva NL Edwards R Sun Y Hrazdina G Dixon RA Stress responses in alfalfa (*Medicago sativa* L.) 11. Molecular cloning and expression of alfalfa isoflavone reductase, a key enzyme of isoflavonoid phytoalexin Plant Mol Biol 1991 17 653 667 1912490 10.1007/BF00037051

[B32] Anterola AM Jeon JH Davin LB Lewis NG Transcriptional control of monolignol biosynthesis in pinus taeda: factors affecting monolignol ratios and carbon allocation in phenylpropanoid metabolism J Biol Chem 2002 277 18272 18280 11891223 10.1074/jbc.M112051200

[B33] Guo DJ Chen F Inoue K Blount JW Dixon RA Downregulation of caffeic acid 3-O-methyltransferase and caffeoyl coa 3-O-methyltransferase in transgenic alfalfa: impacts on lignin structure and implications for the biosynthesis of G and S lignin Plant Cell 2001 13 73 88 11158530 10.1105/tpc.13.1.73 PMC102215

[B34] Doehlemann G Wahl R Horst RJ Voll LM Usadel B Poree F Stitt M Pons-Kühnemann J Sonnewald U Kahmann R Kämper J Reprogramming a maize plant: transcriptional and metabolic changes induced by the fungal biotroph *Ustilago maydis* Plant J 2006 56 181 195 18564380 10.1111/j.1365-313X.2008.03590.x

[B35] Shan XY Zhang YS Peng W Wang ZL Xie DX Molecular mechanism for jasmonate-induction of anthocyanin accumulation in Arabidopsis J Exp Bot 2009 60 3849 3860 19596700 10.1093/jxb/erp223

[B36] Lamblin F Saladin G Dehorter B Cronier D Grenier E Lacoux J Bruyant P Laine E Chabbert B Girault F Overexpression of a heterologous sam gene encoding S-adenosylmethionine synthetase in flax (*Linum usitatissimum*) cells:Consequences on methylation of lignin precursors and pectins Physiol Plantarum 2001 112 223 232 10.1034/j.1399-3054.2001.1120211.x 11454228

[B37] Sanchez-Aguayo I Rodriguez-Galan JM García R Torreblanca J Pardo JM Salt stress enhances xylem development and expression of S -adenosyl-L-methionine synthase in lignifying tissues of tomato plants Planta 2004 220 278 285 15322882 10.1007/s00425-004-1350-2

[B38] Morgan PW Drew MC Ethylene and plant responses to stress Physiol Plantarun 1997 100 620 630

[B39] Kakkar RK Sawhney VK Polyamine research in plants-a changing perspective Physiol Plantarum 2002 116 281 292

[B40] Melida H Caparros-Ruiz D Alvarez J Acebes JL Encina A Deepening into the proteome of maize cells habituated to the cellulose biosynthesis inhibitor dichlobenil Plant Signal Behav 2011 6 143 146 21248490 10.4161/psb.6.1.14304 PMC3122029

[B41] Shoresh M Harman GE The molecular basis of shoot responses of maize seedlings to *trichoderma harzianum* T22 inoculation of the root: a proteomic approach Plant Physiol 2008 147 2147 2163 18562766 10.1104/pp.108.123810 PMC2492612

[B42] Kobayashi I Hakuno H Actin-related defense mechanism to reject penetration attempt by a non-pathogen is maintained in tobacco BY-2 cells Soc Forces 2003 217 340 345 10.1007/s00425-003-1042-3 12728320

[B43] Bednarek P Osbourn A Plant-microbe interactions: chemical diversity in plant defense Science 2009 324 746 748 19423814 10.1126/science.1171661

[B44] Bae H Herman E Bailey B Bae HJ Sicher R Exogenous trehalose alters Arabidopsis transcripts involved in cell wall modification, abiotic stress, nitrogen metabolism, and plant defense Physiol Plantarum 2005 125 114 126

[B45] Breen J Bellgard M Germin-like proteins (GLPs) in cereal genomes: gene clustering and dynamic roles in plant defence Funct Integr Genomics 2010 10 463 476 20683632 10.1007/s10142-010-0184-1

[B46] Vyas D Kumar S Purification and partial characterization of a low temperature responsive Mn-SOD from tea (*Camellia sinensis* (L.) O. Kuntze) Biochem Bioph Res Co 2005 329 831 838 10.1016/j.bbrc.2005.02.051 15752731

[B47] Lee DH KimYS, Lee CB: **The inductive responses of the antioxidant enzymes by salt stress in the rice (*****Oryza sativa *****L.).***J* Plant Physiol 2001 158 737 745

[B48] Ishikawa T Shigeoka S Recent advances in ascorbate biosynthesis and the physiological significance of ascorbate peroxidase in photosynthesizing organisms Biosci Biotech Bioch 2008 72 1143 1154 10.1271/bbb.80062 18460785

[B49] Dixon DP Davis BG Edwards R Functional divergence in the glutathione transferase superfamily in plants J Biol Chem 2002 277 30859 30869 12077129 10.1074/jbc.M202919200

[B50] Manosalva P Davidson R Liu B Zhu X Hulbert S Leung H Leach J A germin-like protein gene family functions as a complex qtl conferring broad-spectrum disease resistance in rice Plant Physiol 2008 149 286 296 19011003 10.1104/pp.108.128348 PMC2613727

[B51] Trapphoff T Beutner C Niehaus K Colditz F Induction of distinct defense-associated protein patterns in *aphanomyces euteiches* (Oomycota)-elicited and -inoculated *medicago truncatula* cell-suspension cultures: a proteome and phosphoproteome approach Mol Plant Microbe In 2009 22 421 436 10.1094/MPMI-22-4-0421 19271957

[B52] Lee J Feng J Campbell KB Scheffler BE Garrett WM Thibivilliers S Stacey G Naiman DQ Tucker ML Pastor-Corrales MA Cooper B Quantitative proteomic analysis of bean plants infected by a virulent and avirulent obligate rust fungus Mol Cell Proteomics 2009 8 19 31 18755735 10.1074/mcp.M800156-MCP200

[B53] Grunwald I Heinig I Thole HH Neumann D Kahmann U Kloppstech K Gau AE Purification and characterisation of a jacalin-related, coleoptile specific lectin from *Hordeum vulgare* Planta 2007 226 225 234 17245569 10.1007/s00425-006-0467-x

[B54] Xiang Y Song M Wei ZY Tong JH Zhang LX Wei ZY Xiao LT Ma ZQ Wang Y A jacalin-related lectin-like gene in wheat is a component of the plant defence system J Exp Bot 2011 62 5471 5483 21862481 10.1093/jxb/err226 PMC3223046

[B55] Liu HY Dai JR Feng DR Liu B Wang HB Wang JF Characterization of a novel plantain *Asr* gene, *MpAsr*, that is regulatedd in response to infection of *Fusarium oxysporum* f. sp. *cubense* and abiotic stresses J Integr Plant Biol 2010 52 315 323 20377692 10.1111/j.1744-7909.2010.00912.x

[B56] Iqbal MJ Yaegashi S Ahsan R Shopinski KL Lightfoot DA Root response to *Fusarium solani* f.sp. *glycines*: temporal accumulation of transcripts in partially resistant and susceptible soybean Theor Appl Genet 2005 110 1429 1438 15815926 10.1007/s00122-005-1969-9

[B57] Grubb CD Abel S Glucosinolate metabolism and its control Trends Plant Sci 2006 11 89 100 16406306 10.1016/j.tplants.2005.12.006

[B58] Margaria P Abba S Palmano S Novel aspects of grapevine response to phytoplasma infection investigated by a proteomic and phospho-proteomic approach with data integration into functional networks BMC Genomics 2013 14 38 23327683 10.1186/1471-2164-14-38 PMC3564869

[B59] Rosenquist M Sehnke P Ferl RJ Sommarin M Larsson C Evolution of the 14-3-3 protein family: does the large number of isoforms in multicellular organisms reflect functional specificity? J Mol Evol 2000 51 446 458 11080367 10.1007/s002390010107

[B60] Finnie C Andersen CH Borch J Gjetting S Christensen AB de Boer AH Thordal-Christensen H Collinge DB Do 14-3-3 proteins and plasma membrane H + −ATPases interact in the barley epidermis in response to the barley powdery mildew fungus? Plant Mol Biol 2002 49 137 147 11999370 10.1023/a:1014938417267

[B61] Wang WX Vinocur B Shoseyov O Altman A Role of plant heat-shock proteins and molecular chaperones in the abiotic stress response Trends Plant Sci 2004 9 244 252 15130550 10.1016/j.tplants.2004.03.006

[B62] Miernyk JA Protein folding in the plant cell Plant Physiol 1999 121 695 703 10557217 10.1104/pp.121.3.695 PMC1539232

[B63] Brown CR Doxsey SJ Hong-Brown LQ Martin RL Welch WJ Molecular chaperones and the centrosome: a role for TCP-1 in microtubule nucleation J Biol Chem 1996 271 824 832 8557692 10.1074/jbc.271.2.824

[B64] Millar AH Heazlewood JL Kristensen BK Braun HP Moller IM The plant mitochondrial proteome Trends Plant Sci 2005 10 36 43 15642522 10.1016/j.tplants.2004.12.002

[B65] Porcel R Aroca R Ruiz-Lozano JM Salinity stress alleviation using arbuscular mycorrhizal fungi: a review Agron Sustain Dev 2012 32 181 200

[B66] Tognetti VB Palatnik JF Fillat MF Melzerc M Hajirezaei MR Valle EM Carrilloa N Functional replacement of ferredoxin by a cyanobacterial flavodoxin in tobacco confers broad-range stress tolerance Plant Cell 2006 18 2035 2050 16829589 10.1105/tpc.106.042424 PMC1533984

[B67] Xiao XW Yang F Zhang S Korpelainen H Li CY Physiological and proteomic responses of two contrasting *Populus cathayana* populations to drought stress Physiol Plantarum 2009 136 150 168 10.1111/j.1399-3054.2009.01222.x 19453505

[B68] Casati P Drincovich MF Edwards GE Andreo CS Malate metabolism by NADP-malic enzyme in plant defense Photosynth Res 1999 61 99 105

[B69] Torres MA Jones JDG Dangl JL Reactive oxygen species signalling in response to pathogens Plant Physiol 2006 141 373 378 16760490 10.1104/pp.106.079467 PMC1475467

[B70] Schaaf J Walter MH Hess D Primary metabolism in plant defense (regulation of a bean malic enzyme gene promoter in transgenic tobacco by developmental and environmental cues) Plant Physiol 1995 108 949 960 12228518 10.1104/pp.108.3.949 PMC157444

[B71] Parker D Beckmann M Zubair H Enot DP Caracuel-Rios Z Overy DP Snowdon S Talbot NJ Draper J Metabolomic analysis reveals a common pattern of metabolic re-programming during invasion of three host plant species by *Magnaporthe grisea* Plant J 2009 59 723 737 19453445 10.1111/j.1365-313X.2009.03912.x

[B72] Finnemann J Schjoerring JK Post-translational regulation of cytosolic glutamine synthetase by reversible phosphorylation and phloem parenchyma 14-3-3 protein interaction Plant J 2000 24 171 181 11069692 10.1046/j.1365-313x.2000.00863.x

[B73] Hidaka T Imai S Hara O Anzai H Murakami T Nagaoka K Seto H Carboxy phosphonoenol pyruvate phosphonomutase, a novel enzyme catalyzing C-P bond formation J Bacteriol 1990 172 3066 3072 2160937 10.1128/jb.172.6.3066-3072.1990 PMC209109

[B74] Verniquet F Gaillard J Neuburger M Douce R Rapid inactivation of plant aconitase by hydrogen peroxide Biochem J 1991 276 643 648 1648348 10.1042/bj2760643 PMC1151053

[B75] Nunes-Nesi A Carrari F Lytovchenko A Smith AMO Loureiro ME Ratcliffe RG Sweetlove LJ Fernie AR Enhanced photosynthetic performance and growth as a consequence of decreasing mitochondrial malate dehydrogenase activity in transgenic tomato plants Plant Physiol 2005 137 611 622 15665243 10.1104/pp.104.055566 PMC1065362

[B76] Sobhanian H Razavizadeh R Nanjo Y Ehsanpour AA Jazii FR Motamed N Komatsu S Proteome analysis of soybean leaves, hypocotyls and roots under salt stress Proc Natl Acad Sci U S A 2010 8 19 10.1186/1477-5956-8-19 PMC2859372 20350314

[B77] Zorb C Schmitt S Muhling KH Proteomic changes in maize roots after short-term adjustment to saline growth conditions Proteomics 2011 10 4441 4449 21136597 10.1002/pmic.201000231

[B78] Rolland F Moore B Sheen J Sugar sensing and signaling in plants Plant Cell 2002 14 185 205 10.1105/tpc.010455 PMC151255 12045277

[B79] Ganapathi TR Suprasanna P Bapat VA Rao PS Propagation of Banana through encapsulated shoot tips Plant Cell Rep 1992 11 571 574 24213289 10.1007/BF00233095

[B80] Isaacson T Damasceno CMB Saravanan RS He YH Catala C Saladie M Rose JKC Sample extraction techniques for enhanced proteomic analysis of plant tissues Nat Protoc 2006 1 769 774 17406306 10.1038/nprot.2006.102

[B81] MM B A rapid and sensitive method for the quantitation of microgram quantities of protein utilizing the principle of protein-dye binding Anal Biochem 1976 72 248 254 942051 10.1016/0003-2697(76)90527-3

[B82] Neuhoff V Arold N Taube D Ehrhardt W Improved staining of proteins in polyacrylamide gels including isoelectric focusing gels with clear background at nanogram sensitivity using Coomassie Brilliant Blue G-250 and R-250 Electrophoresis 1998 9 255 262 2466658 10.1002/elps.1150090603

[B83] Guo HX Zhang HZ Li YC Ren JP Wang X Niu HB Yin J Identification of changes in wheat (*Triticum aestivum* L.) seeds proteome in response to anti–trxs gene Plos One 2011 6 e222555 10.1371/journal.pone.0022255 PMC3139615 21811579

[B84] Li YF Zhang ZH Nie YF Zhang LH Wang ZZ Proteomic analysis of salicylic acid-induced resistance to *Magnaporthe oryzae* in susceptible and resistant rice Proteomics 2012 12 2340 2354 22730241 10.1002/pmic.201200054

[B85] Podevin N Krauss A Henry I Swennen R Remy S Selection and validation of reference genes for quantitative RT-PCR expression studies of the non-model crop Musa Mol Breeding 2012 30 1237 1252 10.1007/s11032-012-9711-1 PMC3460175 23024595

