# Supplementary material for: Proteomic analysis of Fusarium oxysporum f. sp. cubense tropical race 4-inoculated response to Fusarium wilts in the banana root cells
Source: Proteome Sci. 2013 Sep 26;11:41. doi: 10.1186/1477-5956-11-41 (PMC3850410; doi:10.1186/1477-5956-11-41)
Supplement: Additional file 1: Table S1 — Identification of differentially expressed proteins from banana root inoculated with Foc4 by MALDI-TOF/TOF MS. [file 1477-5956-11-41-S1.doc]

**Additional file**

**Table S1** Identification of differentially expressed proteins from banana root inoculated with FOC4 by MALDI-TOF/TOF MS

| Protein spot no. | Protein name | Accession number | organism | Theoretical  PI/MW | scoring peptides | Protein score | Protein score  C.I.% | Sequence coverage  (%) | Chang fold | | |
| --- | --- | --- | --- | --- | --- | --- | --- | --- | --- | --- | --- |
|  |  |  |  |  |  |  |  |  | Brazil | Nongke No.1 | Yueyoukang I |
| Defense | | | | | | | | | | | |
| 2 | Pathogenesis-related protein 1 | GSMUA_Achr3P08250_001 | *Musa acuminata* | 5.42/17195.9 | FLPGVEDNEGEIVK(67)/QLNFSPAIPFGYVK(92)/LETASTHFKFEPK(59)/LDFVDVDKLECK(43) | 319 | 100 | 46 |  | | |
| 16 | RecName: Full= Alpha-amylase inhibitor/endochitinase | gi|266324 | *Coix lacryma-jobi* | 6.07/14637.7 | GPIQISXNYNYGPAGR(77) | 86 | 99.719 | 12 |  | | |
| **Secondary metabolism** | | | | | | | | | | | |
| 28 | Caffeoyl-CoA O-methyltransferase | GSMUA_Achr6P36400_001 | *Musa acuminata* | 5.21/27809.2 | DFVLELNNALAADPR(127)/SLLQSDELYQYILETTVYPR(157)/ENYEIGLPVIQK(52)/IEICQLPVGDGVTLCR(109)/ILAMDINRENYEIGLPVIQK(72)/VGGVIAYDNTLWSGSVVAPPDAPMR(53) | 685 | 100 | 40 |  | | |
| 32 | Isoflavone reductase homolog | GSMUA_Achr2P14320_001 | *Musa acuminata* | 6.1/34070 | YLPSEFGNDVDR(104)/LLSDFQAAGVTLVQGDIY(122)/FIVFASAR(61)/LGNPTFALVR(79)/ILIIGGTGYIGK(51)/YTTVDEFLNR(86)/RYLPSEFGNDVDR(51)/AIFLNEDDIGTYTIK(96)/YLPSEFGNDVDRSHAVEPAK(82) | 934 | 100 | 75 |  | | |
| 34 | Leucoanthocyanidin dioxygenase | GSMUA_Achr5P04080_001 | *Musa acuminata* | 5.61/40934.2 | IVLKPLEELVADGTPAK(86)/IVLKPLEELVADGTPAKFPPR(68)/ACVEEVRK(17)/TFEQHIQHK(48)/VGKEFFDLPIEQK(56)/SGINEIPTEYVRPESER(46)/AAEGPQIPVVDLQGFDSPDEEVRR(36) | 501 | 100 | 46 | +^*^ | | |
| 36 | S-adenosylmethionine synthase | GSMUA_Achr8P06200_001 | *Musa acuminata* | 5.48/43651.9 | FVIGGPHGDAGLTGR(81)/VLVNIEQQSPDIAQGVHGHFTK(71)/TAAYGHFGR(8)/TQVTVEYR(8)/NDHGAMIPVR(11)/TIFHLNPSGR(48) | 314 | 100 | 36 | +* | | |
| **The synthesis of polysaccharose** | | | | | | | | | | | |
| 42 | Alpha-1,4-glucan-protein synthase | GSMUA_Achr4P23520_001 | *Musa acuminata* | 7.57/30976.7 | GTLFPMCGMNLAFDR(109)/NLLSPATPHFFNTLYDPYR(72)/GTLFPMCGMNLAFDR(54)/ELIGPAMYFGLMGDGQPIGR(28) | 298 | 100 | 57 |  | | |
| **Cell cytoskeleton** | | | | | | | | | | | |
| 37 | Actin | GSMUA_Achr8P02660_001 | *Musa acuminata* | 5.31/41820.1 | SYELPDGQVITIGAER(150)/VAPEEHPILLTEAPLNPK(102)/AGFAGDDAPR(76)/GYSFTTTAER(64)/AVFPSIVGRPR(56)/GEYEESGPAIVHR(98)/TTGIVLDSGDGVSHTVPIYEGYALPHAILR(99) | 807 | 100 | 54 |  | | |
| **Oxidative-redox stress** | | | | | | | | | | | |
| 11 | Germin-like protein 12-1 | GSMUA_Achr9P30640_001 | *Musa acuminata* | 5.42/17195.9 | LPGLNTLGVAMSR(111)/LPGLNTLGVAMSR(57) | 319 | 100 | 16 | +^*^ | | |
| 12 | Germin-like protein 12-1 | GSMUA_Achr9P30640_001 | *Musa acuminata* | 5.67/25231.9 | LPGLNTLGVAMSR(98)/LPGLNTLGVAMSR(38) | 103 | 100 | 5 | +^*^ | | |
| 13 | Germin-like protein 12-1 | GSMUA_Achr9P30640_001 | *Musa acuminata* | 5.67/25231.9 | GDAFVFPR(52)/IDYAPFGLNPPHSHPR(126)/KGDAFVFPR(51) | 251 | 100 | 19 |  | | |
| 14 | Protein IN2-1 homolog B | GSMUA_Achr6P33160_001 | *Musa acuminata* | 6/31726.2 | IELVPVDLLNRPAWYK(89)/LASLPPSLTAASEPPALFDGTTR(182)/YIDAHFEGPALKPDDPAK(50)/VPSLEHNNEVRGESLDLIK(51)/IELVPVDLLNRPAWYKDK(23) | 462 | 100 | 39 |  | | |
| 15 | Germin-like protein 12-1 | GSMUA_Achr9P30640_001 | *Musa acuminata* | 5.67/25231.9 | KGDAFVFPR(72)/IDYAPFGLNPPHSHPR(99)/GDAFVFPR(44)/TTVGWLQQQQWLDIAQEYGQR(13) | 258 | 100 | 19 |  | | |
| 29 | L-ascorbate peroxidase | GSMUA_Achr5P07280_001 | *Musa acuminata* | 5.31/27470.9 | YAADEDAFFADYTEAHLK(127)/DVFGHMGLSDQDIVALSGGHTLGR(145)/TGGPFGTMR(50)/TGGPFGTMR(24)/NCAPLMLR(53)/NCAPLMLR(26)/EDKPEPPVEGR(81)/ALLTDPVFRPLVEK(96)/DVFGHMGLSDQDIVALSGGHTLGR(106) | 675 | 100 | 65 |  | | |
| 30 | Probable glutathione S-transferase GSTF1 | GSMUA_Achr2P02330_001 | *Musa acuminata* | 5.71/23886.2 | NPFGQVPAFEDGDLMLFESR(114)/NPFGQVPAFEDGDLMLFESR(50) | 120 | 100 | 9 |  | | |
| 31 | Superoxide dismutase [Mn] 3.1 | GSMUA_Achr8P21350_001 | *Musa acuminata* | 7.09/26441.5 | KLCVETTANQDPLVTK(118)/HHQAYVTNYNNALEQLETAVAK(176)/NVRPDYLK(35) | 381 | 100 | 31 |  | | |
| **Signal conduction** | | | | | | | | | | | |
| 1 | Putative horcolin | GSMUA_Achr9P10720_001 | *Musa acuminata* | 5.1/18770.3 | INAGDIIDAIEITFTR(166)/TFGPFGAYEGTPFSIPVAEGK(88)/RTFGPFGAYEGTPFSIPVAEGK(45)/LTLTTNR(34) | 416 | 100 | 44 | +^*^ | | |
| 8 | Abscisic stress-ripening protein 3 | GSMUA_Achr6P25740_001 | *Musa acuminata* | 6.43/11788.8 | HKEHLGELGALAAGAYALHEK(104)/VAEEIAATVAVGSAGFAFHEHHEK(68) | 222 | 100 | 50 |  | | |
| 33 | Abscisic stress ripening protein | GSMUA_Achr11P12030_001 | *Musa acuminata* | 5.96/16206.7 | IEEEIAAAVAVGSGGYAFHEHHEK(181)/HKIEEEIAAAVAVGSGGYAFHEHHEK(189)/HHHHLF(32)/KHHHHLF(46)/HHHHLFHHHK(68)/HKEHLGEMGAVAAGAFALYEK(116) | 771 | 100 | 60 |  | | |
| 35 | Auxin-induced protein PCNT115 | GSMUA_Achr8P26850_001 | *Musa acuminata* | 5.52/38835.9 | YIGLCEASPDTIR(105)/AGTEGLPEGSIVALNPR(144)/DIEDEIIPLCR(88)/LGVDYIDLYFPHR(91)/YIGLCEASPDTIRR(75)/ELGIGVIAYSPLGHGFFAGR(92) | 719 | 100 | 44 |  | | |
| 38 | 14-3-3-like protein GF14-E | GSMUA_Achr7P10320_001 | *Musa acuminata* | 4.54/14728.3 | SAQDIALAELAPTHPIR(139) | 193 | 100 | 66 |  | | |
| 39 | 14-3-3-like protein GF14-6 | GSMUA_AchrUn_randomP24510_001 | *Musa acuminata* | 4.53/14888.4 | SAQDIALAELAPTHPIR(85) | 106 | 100 | 27 |  | | |
| **Molecular chaperones** | | | | | | | | | | | |
| 9 | Heat shock 22 kDa protein | GSMUA_Achr10P30370_001 | *Musa acuminata* | 6.02/24039.3 | RPDSPAAAHR(83)/DYDDDNRDVDLER(79)/IDLPPESYR(66)/EDEHALHLR(65)/GWDAREDEHALHLR(58)/VWAEHNTLVIK(34) | 482 | 100 | 58 |  | | |
| 10 | Heat shock 22 kDa protein | GSMUA_Achr10P30370_001 | *Musa acuminata* | 6.02/24039.3 | GWDAREDEHALHLR(45)/SLSQVLNLMDQMLDNPIFAAAGPSGAAGGLR(57)/IDLPPESYR(42)/EDEHALHLR(42) | 247 | 100 | 43 |  | | |
| 22 | T-complex protein 1 subunit gamma | GSMUA_Achr6P23290_001 | *Musa acuminata* | 6.04/61135.9 | TAIEAACMLLR(42)/TLAQNCGVNVIR(51)/NYHPTVICR(24)/MLLDAAGGIVVTNDGNAILR(33)/ACGAVVVNRPEELQESDIGTGAGLFEIK(19) | 238 | 100 | 30 |  | | |
| 26 | Protein disulfide-isomerase | GSMUA_Achr1P16970_001 | *Musa acuminata* | 4.77/56623.6 | DYIDGNLKPYR(77)/LSQYDGDRTAEAIINFVK(73)/GDPTVKLPVVR(58)/LRTEYDFGHTSEAK(37) | 539 | 100 | 49 |  | | |
| 40 | late embryogenesis abundant protein | GSMUA_Achr10P04300_001 | *Musa acuminata* | 4.85/20567.6 | VALIVDVPIFGR(72)/TGEIPIPYKPDVDIEK(110)/MEIPISFRPK(39)/MEIPISFRPK(26)/FSFEETIANLHLK(51) | 346 | 100 | 50 |  | | |
| **Energy** | | | | | | | | | | | |
| 21 | Ferredoxin-NADP reductase | GSMUA_Achr4P31670_001 | *Musa acuminata* | 8.31/42786.5 | IMLLPEEDPNATHIMIATGTGVAPFR(90)/IMLLPEEDPNATHIMIATGTGVAPFR(133) | 160 | 100 | 23 |  | | |
| **Primary metabolism** | | | | | | | | | | | |
| 17 | Glutamine synthetase nodule isozyme | GSMUA_Achr1P22290_001 | *Musa acuminata* | 5.62/39606.6 | HETADINTFVWGVANR(112) | 125 | 100 | 12 |  | | |
| 18 | Glutamine synthetase nodule isozyme | GSMUA_Achr1P22290_001 | *Musa acuminata* | 5.62/39606.6 | HKEHIAAYGEGNER(108)/HETADINTFVWGVANR(100) | 274 | 100 | 24 |  | | |
| 19 | Glutamine synthetase nodule isozyme | GSMUA_Achr3P20250_001 | *Musa acuminata* | 5.73/39227.6 | HKEHIAAYGEGNER(61)/HETADINTFLWGVANR(103)/IIAEYIWIGGSGLDIR(61)/RPASNMDPYVVTSMIAETTIIGK(19)/GNNILVMCDCYTPAGEPIPTNKR(15) | 311 | 100 | 26 |  | | |
| 20 | Putative carboxyvinyl-carboxyphosphonate phosphorylmutase | GSMUA_Achr6P25770_001 | *Musa acuminata* | 5.76/33411.1 | AGFVSGYAVSASR(73)/EAIGDADFFLIAR(20)/TYNETGAR(14)/LGMPDIGLLTPPEMADAAR(14) | 163 | 100 | 27 |  | | |
| 23 | NADP-dependent malic enzyme | GSMUA_AchrUn_randomP19370_001 | *Musa acuminata* | 5.98/65328.8 | AYDLGLATR(63)/SIQVIVVTDGER(79)/GLIYPPFSNIR(49)/YAESCMYTPVYR(54) | 346 | 100 | 27 |  | | |
| 24 | Putative aconitate hydratase | GSMUA_Achr9P30280_001 | *Musa acuminata* | 7.59/110205.1 | SENAVQANMEFEFQR(121)/YKADGHDTIVLAGAEYGSGSSR(93)/ILLESAIR(23)/DFNSYGSR(53)/IDRLPYSIR(25)/LVEIPFKPAR(73)/FDFHGQPAELK(53)/YYSLPALNDPR(60)/MFVDYTEPQKER(39)/INPLVPVDLVIDHSVQVDVAR(84) | 777 | 100 | 30 |  | | |
| 25 | Putative aconitate hydratase | GSMUA_Achr9P30280_001 | *Musa acuminata* | 7.59/110205.1 | LVEIPFKPAR(52)/FDFHGQPAELK(31)/ILLESAIR(23)/DFNSYGSR(21)/YYSLPALNDPR(31) | 226 | 100 | 18 |  | | |
| 27 | Fructokinase-1 | GSMUA_Achr4P16250_001 | *Musa acuminata* | 5.06/33634.5 | APGGAPANVAIAVAR(122)/ENGVDDTGVTFDAGAR(128)/LGDDEFGR(57)/TALAFVTLR(70)/LPLWTSPEEAR(76)/EAGALLSYDPNLR(77)/ADGEREFMFYR(18)/KGAIPSLPNVAEAMR(13)/AAIFHYGSISLITEPCR(38) | 855 | 100 | 61 |  | | |
| 41 | Fructokinase-2 | GSMUA_Achr11P11150_001 | *Musa acuminata* | 4.94/35429.2 | APGGAPANVAIAVAR(131)/DNGVDDSGVTFDAGAR(125)/EFMFYR(35)/LGDDEFGR(57)/TALAFVTLR(71)/LPLWPSAAEAR(93)/EAGALLSYDPNLR(109)/VFHYGSISLITEPCR(116) | 944 | 100 | 66 |  | | |
| 43 | Malate dehydrogenase | GSMUA_Achr4P21920_001 | *Musa acuminata* | 6/35814.4 | KLSSALSAASSACDHIR(155)/NVIIWGNHSSTQYPDVSHATVK(156)/ALGQISER(37)/TPSGEKPVR(67)/LNVQVSDVK(64)/GEFITTVQQR(69)/MELVDAAFPLLK(70)/SQASALEAYAAPNCK(86)/LSSALSAASSACDHIR(51)/VLVTGAAGQIGYALVPMIAR(60) | 963 | 100 | 53 |  | | |
| **Unknown** | | | | | | | | | | | |
| 3 |  | gi|407351756 | *Musa acuminata* | 5.48/23879.5 | IIPEYFAGAELVGDGEAGSTK(147)/TKTEYDTIDDAPLPEDEVQK(144) | 808 | 100 | 55 |  | | |
| 4 |  | gi|407348852 | *Musa acuminata* | 5.4/15519.7 | DHVEVLDHGSHTMR(134)/TKTEYDTIDDAPLPEDEVQK(148) | 585 | 100 | 61 |  | | |
| 5 |  | gi|182664797 | *Musa acuminata* | 9.72/26310.4 | SGGGGGYGGGGYGGGGGGR(45) | 70 | 97.526 | 8 |  | | |
| 6 |  | gi|407351604 | *Musa acuminata* | 6.04/12114.1 | NHVEVLDHGSHTLK(123)/IMPQYFSGAELIGDGEAGSTK(117) | 417 | 100 | 57 |  | | |
| 7 |  | gi|407348788 | *Musa acuminata* | 6.1/14338.3 | NHVEVLDHGSHTLK(125)/IMPQYFSGAELIGDGEAG(115) | 510 | 100 | 66 |  | | |

“+*”: represent qualitative changed protein spots
